# First person – Niki Jalava

**DOI:** 10.1242/bio.060525

**Published:** 2024-05-29

## Abstract

First Person is a series of interviews with the first authors of a selection of papers published in Biology Open, helping researchers promote themselves alongside their papers. Niki Jalava is first author on ‘ Short- and long-term exposure to high glucose induce unique transcriptional changes in osteoblasts *in vitro*’, published in BiO. Niki is a doctoral researcher in the lab of Kaisa Ivaska at University of Turku, Institute of Biomedicine, Finland, investigating hyperglycemia and obesity on the bone-secreted proteins and intertissue communication.


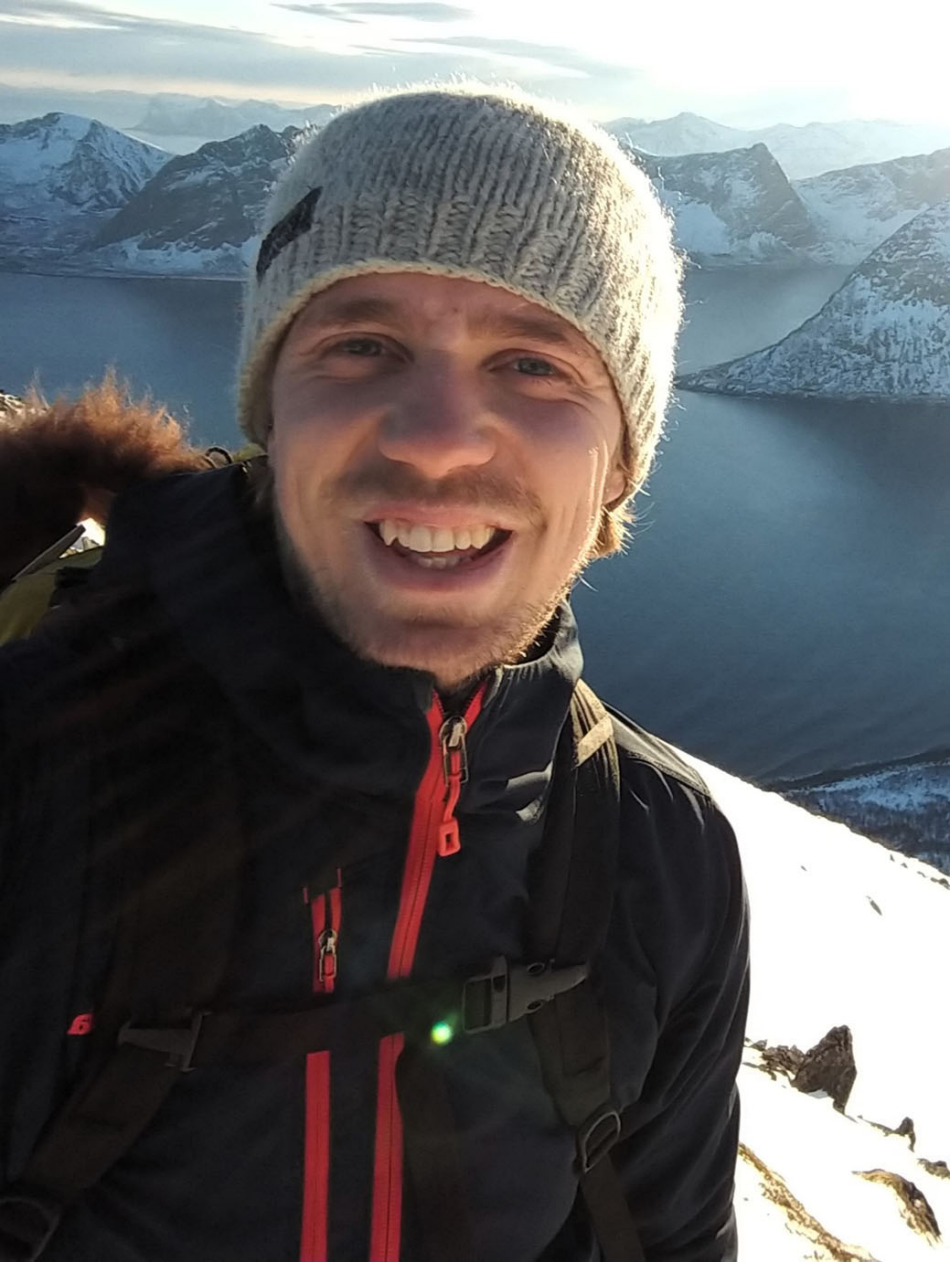


**Niki Jalava**

**Describe your scientific journey and your current research focus**

This was my first published article as a first author, and certainly one I will remember for the rest of my life. During my Master's studies, I focused on bioactive materials and novel bone implant manufacturing and developed a strong interest for research and development. After graduating, I had the opportunity to pursue a PhD in the bone research lead by Adj. Prof. Kaisa Ivaska. Currently, I am focusing on the role of hyperglycemia and obesity on the bone-secreted proteins and intertissue communication. For example, how metabolic disorders, such as type 2 diabetes, affect the bones, and vice versa.

**Who or what inspired you to become a scientist?**

I have always wanted to contribute to society in an impactful way and science lets me do that in manner that is both exciting and fulfilling. Curiosity for natural sciences, health, and biology combined with the constant will to improve makes scientist's career a natural choice for me.

**How would you explain the main finding of your paper?**

We tested how exposure to high glucose environment affects bone-forming osteoblasts' gene expression. Utilizing RNA-sequencing, we studied all the osteoblasts' active genes in physiological (5.5 mM) and high glucose (25 mM) environments. We discovered that short-term 24 h exposure induced a transient stimulatory effect on bone formation, mineralization, and bone matrix remodelling. Long-term, 10-day exposure instead had a negative effect on osteoblast function, which we believe is due to increased oxidative stress and inability to neutralise reactive oxygen species.

**What are the potential implications of this finding for your field of research?**

Recognition of the bone as a target for metabolic complications has the potential to find new drug targets or repurpose current bone-targeting drugs for treatment of metabolic disorders.

**Figure:**
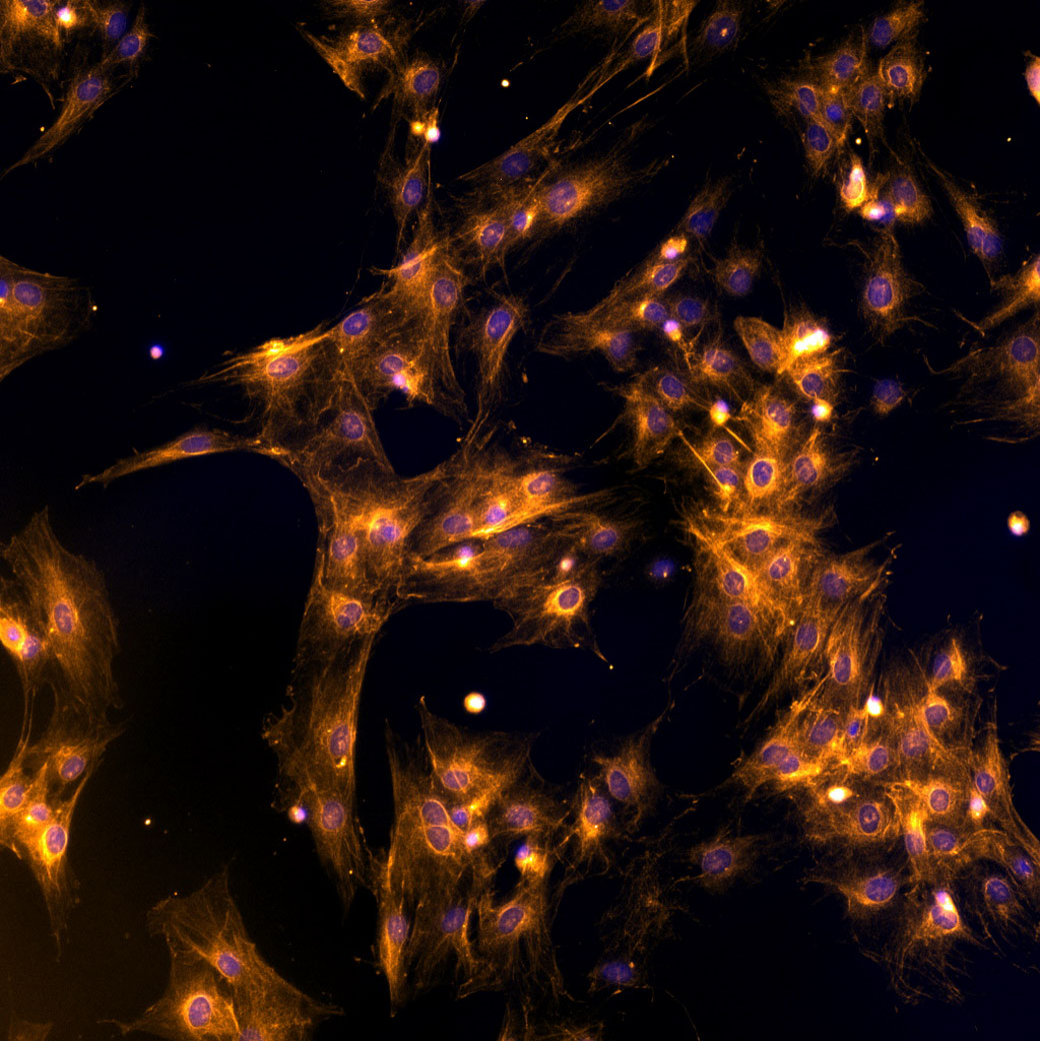
Overexposed unspecific antibody binding reveals beautiful intracellular structures in bone marrow stromal cells.

**Which part of this research project was the most rewarding?**

The most rewarding part for me was the learning of new techniques and technologies required to complete a full research article. I was surprised by the number of skills that are needed to bring a research project to the end – all the way from cell isolation and culturing to literature review and writing, without forgetting collaboration and self-leadership skills.

**What do you enjoy most about being an early-career researcher?**

Having the freedom to plan my own days and discuss research with my colleagues. Every day is a learning experience, and this job always provides me with ways to improve myself.

Submit an abstract to a conference, agree to a small presentation to a local community, visit networking events.

**What piece of advice would you give to the next generation of researchers?**

Learn to recognize opportunities for personal and career growth. Submit an abstract to a conference, agree to a small presentation to a local community, visit networking events. It might be scary at first to give a public presentation or talk to strangers, but from experience I can say it is worth the effort. Put yourself out there and have an open mind.

**What's next for you?**

After finishing my PhD, I will most likely find a position in the industrial or public sector that allows me to use my expertise and knowledge to help people in need.
